# Protective Effects of Black Cumin (*Nigella sativa*) and Its Bioactive Constituent, Thymoquinone against Kidney Injury: An Aspect on Pharmacological Insights

**DOI:** 10.3390/ijms22169078

**Published:** 2021-08-23

**Authors:** Md. Abdul Hannan, Md. Sarwar Zahan, Partha Protim Sarker, Akhi Moni, Hunjoo Ha, Md Jamal Uddin

**Affiliations:** 1 ABEx Bio-Research Center, East Azampur, Dhaka 1230, Bangladesh; hannanbmb@bau.edu.bd (M.A.H.); mszahan@hotmail.com (M.S.Z.); sarkerpartha124124@gmail.com (P.P.S.); akhimoni840818@gmail.com (A.M.); 2 Department of Biochemistry and Molecular Biology, Bangladesh Agricultural University, Mymensingh 2202, Bangladesh; 3 Graduate School of Pharmaceutical Sciences, College of Pharmacy, Ewha Womans University, Seoul 120-750, Korea; hha@ewha.ac.kr

**Keywords:** black cumin, kidney injury, nephrotoxicity, thymoquinone, xenobiotic stress

## Abstract

The prevalence of chronic kidney disease (CKD) is increasing worldwide, and a close association between acute kidney injury (AKI) and CKD has recently been identified. Black cumin (*Nigella sativa*) has been shown to be effective in treating various kidney diseases. Accumulating evidence shows that black cumin and its vital compound, thymoquinone (TQ), can protect against kidney injury caused by various xenobiotics, namely chemotherapeutic agents, heavy metals, pesticides, and other environmental chemicals. Black cumin can also protect the kidneys from ischemic shock. The mechanisms underlying the kidney protective potential of black cumin and TQ include antioxidation, anti-inflammation, anti-apoptosis, and antifibrosis which are manifested in their regulatory role in the antioxidant defense system, NF-κB signaling, caspase pathways, and TGF-β signaling. In clinical trials, black seed oil was shown to normalize blood and urine parameters and improve disease outcomes in advanced CKD patients. While black cumin and its products have shown promising kidney protective effects, information on nanoparticle-guided targeted delivery into kidney is still lacking. Moreover, the clinical evidence on this natural product is not sufficient to recommend it to CKD patients. This review provides insightful information on the pharmacological benefits of black cumin and TQ against kidney damage.

## 1. Introduction

Kidney diseases are considered as a global public health problem. Chronic kidney disease (CKD) is a critical regulator of morbidity and mortality from non-communicable diseases, while the incidence rate of acute kidney injury (AKI) is increasing worldwide [1]. Patients with a history of AKI may develop CKD [2,3]. The pathophysiology of kidney disease is complex and includes inflammation, tubular injury, and vascular damage [4,5]. Being excretory organs, kidneys are particularly vulnerable to the toxic effects of xenobiotics and their metabolites. With the increasing exposure to xenobiotics such as drugs, toxins, and environmental chemicals, the global incidence of chronic human diseases including kidney disease is growing at an alarming rate [6]. Xenobiotics impair the structural and functional capacity of kidneys by inducing oxidative stress, inflammation, apoptosis, and fibrosis, leading to the development of AKI and CKD [6,7]. Although the pathophysiology of various kidney diseases has been studied, many targeted clinical therapies have failed [8]. Thus, urgent interventions are needed to treat patients with kidney disease. 

Black cumin (*Nigella sativa* L.) is a popular spicy herb and its seeds, in particular, have traditionally been indicated in the management of various human ailments, including those affecting the renal system [9]. Thymoquinone (TQ), the main active component of black cumin seed and its oil, was shown to promote the function of different vital organs, including kidney function [10]. Mounting evidence shows that black cumin and TQ can alleviate kidney complications caused by various stress factors, namely chemotherapeutic agents, metabolic deficits, and environmental toxicants [11]. Evidence from the preclinical studies has shown that black cumin seed (in the form of powder, extracts, or oil) and TQ protect against kidney injuries induced by ischemia [12,13], cancer chemotherapeutic drugs (methotrexate and cisplatin) [14,15], analgesics (paracetamol, acetylsalicylic acid and aspirin) [16,17,18], heavy metal (arsenic and cadmium) [19,20], pesticide (piconazole and diazinon) [21,22], and other chemicals (carbon tetrachloride and sodium nitrite) [23,24]. Evidence, athough limited, also suggests clinical improvements in CKD patients treated with black cumin [25,26,27]. Besides, black cumin was shown to be effective in modifying various risk factors for kidney disease such as hypertension, atherosclerosis, dyslipidemia, hyperglycemia, and diabetes [11]. The kidney-protective effects of black cumin are owing to its antioxidant, anti-inflammatory, immunomodulatory, antiapoptotic, and antifibrotic properties [11,28,29]. In this review, we provide a brief account of the protective effects of black cumin against various kidney injuries and discuss molecular mechanisms where possible. 

## 2. Methodology

Online scientific databases, such as PubMed, Google Scholar, Scopus, and the Web of Science were searched to retrieve literature using keywords, including black cumin, *N. sativa*, essential oil, thymoquinone, kidney injury, oxidative stress, inflammation, fibrosis, nephrotoxicity, and xenobiotic stress. Both preclinical and clinical studies have been documented. Literature published in languages other than English was excluded from this review. All figures were generated using Microsoft Powerpoint.

## 3. Antioxidant and Anti-Inflammatory Effects of Black Cumin and TQ

**Figure 1:**
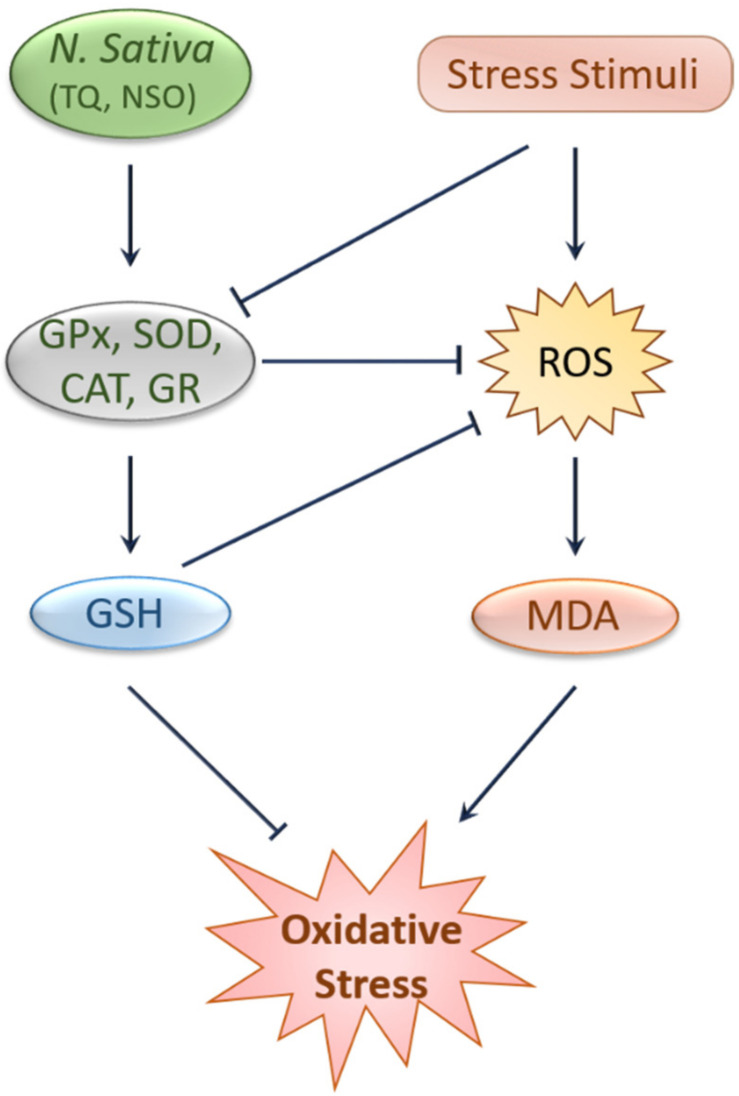
Protection against oxidative stress by black cumin and its constituents. Stress stimuli like CP and chlorpyrifos reduce antioxidant enzymes and elevate ROS and MDA levels, leading to oxidative stress, which was attenuated by *N*. *sativa* and TQ through a mechanism involving the upregulation of antioxidants enzymes and molecules, such as GPx, GR, SOD, CAT, and GSH and the subsequent reduction of ROS and MDA levels. CAT, Catalase; GPx, Glutathione peroxidase; GSH, Glutathione; GR, Glutathione reductase; MDA, Malondialdehyde; NSO, *N. sativa* oil; ROS, Reactive oxygen species; SOD, Superoxide dismutase; TQ, Thymoquinone.

**Figure 2:**
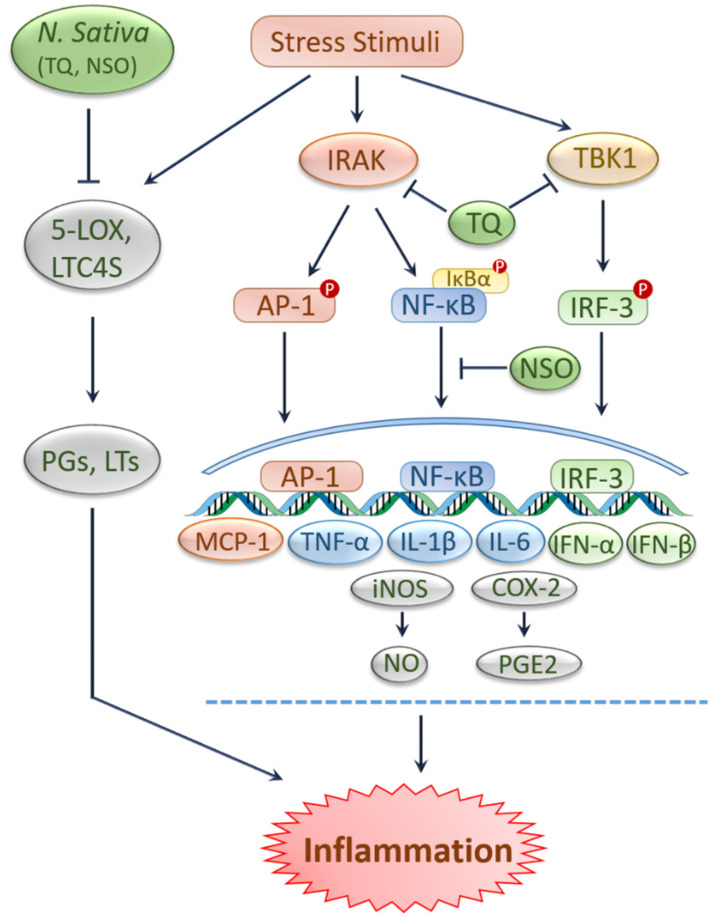
Protection against inflammation by black cumin and its constituents. Stimulation of various extrinsic and intrinsic stressors triggers inflammatory signals. Activity of inflammatory enzymes such as 5-LOX and LTC4S resulted in the generation of leukotrienes and prostaglandins, respectively, leading to inflammation. NSO and TQ prevent inflammation by inhibiting 5-LOX and LTC4S. NSO reduces inflammation by downregulating IL-6. TQ suppresses pro-inflammatory cytokines by inhibiting AP-1/NF-κB pathways. TQ inhibits TBK1 and lowers IFN expression by downregulating IRF-3. AP-1, Activated protein-1; 5-LOX, 5-lipoxygenase; IFN-α, Interferon alfa; IFN-β, Interferon beta; IL-1β, Interleukin-1 beta; IL-6, Interleukin-6; IRF-3, Interferon regulatory factor 3; IRAK, interleukin-1 receptor-associated kinase; LTC4S, leukotriene C4 synthase; LTs, leukotrienes; MCP-1, monocyte chemoattractant protein 1; NF-κB, Nuclear factor-kappa B; NO, nitric oxide; NSO, *N*. *sativa* oil; PGs, prostaglandins; PGE2, Prostaglandin E2, TBK1, TANK-binding kinase 1; TNF-α, Tumor necrosis factor-alpha; TQ, thymoquinone; COX-2, cyclooxygenase 2; iNOS, nitric oxide synthase.

Oxidative stress and inflammation are two pathogenic events that are known to be crucially implicated in the pathobiology of various kidney problems, including kidney toxicity, AKI, and CKD [30,31]. Many natural products have proven potential in alleviating oxidative stress and inflammation [32,33] and have thereby shown efficacy against kidney diseases (Figure 1 and Figure 2).

Substantial evidence from animal and human studies have confirmed the protective effects of black cumin and TQ against oxidative stress [28,34,35,36,37,38]. Black cumin upregulated erythrocyte glutathione peroxidase (GPx), glutathione-S-transferase (GST), and superoxide dismutase (SOD) levels and simultaneously lowered plasma malondialdehyde (MDA) levels [38,39]. In two similar studies, black cumin increased the level of antioxidant enzymes, such as SOD and catalase (CAT), and antioxidant molecules, such as glutathione (GSH) and decreased reactive oxygen species (ROS) [40,41]. Moreover, *N. sativa* oil (NSO) reduced chlorpyrifos-induced oxidative stress by decreasing ROS and nitrous oxide production in the Wister rats model [42]. Daily intake of TQ (5 mg/kg) for five weeks elevated CAT, glutathione reductase (GR), GPx, SOD, and GSH level in liver tissues [43]. Similarly, TQ elevates SOD, CAT, and GSH levels, upregulates antioxidant genes, and downregulates pro-oxidant genes [44]. Another study in rabbits revealed that consuming black cumin seeds (600 mg/kg) decreased MDA and increased total antioxidant levels in the blood [45]. Again, combined supplementation of TQ and NSO exhibited antioxidant capabilities against cisplatin (CP)-induced abnormalities [46]. One meta-analysis report on black cumin seed showed enhanced SOD levels without any visible effect on MDA level and total antioxidant capacity [47]. Even so, this preclinical evidence of the antioxidant effects of black cumin has been elaborated in clinical studies. Combined ingestion of black cumin seed and *Allium sativum* over eight weeks improved antioxidant status in 30 postmenopausal, healthy women [39]. Again, supplementation of NSO and a low-calorie diet showed an improvement in antioxidant status in a clinical trial of 50 obese volunteers [48].

Along with protection against oxidative stress, black cumin and TQ have been shown to curb inflammation as claimed by previous literature [9,28,35,49]. The extracts and bioactive compounds of black cumin, such as TQ, nigellone, and α-hederin revealed anti-histaminic, anti-immunoglobulin, anti-leukotrienes, anti-eosinophilic, and anti-inflammatory effects in several models [50]. In addition, TQ suppressed pro-inflammatory factors such as nitric oxide (NO), nitric oxide synthase (iNOS), tumor necrosis factor-alpha (TNF-α), interleukin-1 beta (IL-1β), interleukin-6 (IL-6) and cyclooxygenase 2 (COX-2) by inhibiting IRAK-linked AP-1/NF-κB pathways [51]. In human blood cells, NSO and TQ inhibited 5-lipoxygenase (5-LOX) and leukotriene C4 synthase (LTC4S) [52], which may generate inflammatory mediators like leukotrienes and prostaglandins [52,53]. In another study, TQ inhibited TANK-binding kinase 1 (TBK1), lowered the type I interferons (IFN) mRNA expression and downregulated the interferon regulatory factor 3 (IRF-3) signaling pathways in lipopolysaccharides (LPS)-stimulated murine macrophage-like RAW264.7 cells [54]. In lung tissue, NSO treatment caused a reduction in IgG1, IgG2a, interleukin-2 (IL-2), interleukin-12 (IL-12), interleukin-10 (IL-10), IFN-γ levels and inflammatory cells [55]. Additionally, administration of NSO significantly reduced IL-6, slightly reduced IL-12, and TNF-α levels in rats affected with carrageenan-induced paw edema [56]. Similarly, supplementation of 10% NSO alleviated inflammation in paw edema rats with a lessened leucocytes count and TNF-α level [49]. Again, an experiment in human pre-adipocytes demonstrated that the fresh extracted and stored NSO resulted in decreased IL-6 and IL-1β levels, respectively [57]. 

## 4. Protective Effects of Black Cumin and TQ against Kidney Injury

Black cumin and TQ have been reported to alleviating various abnormalities that often interfere with the physiological function of kidneys. In the following sections, the kidney-protective effects of black cumin and TQ are discussed, highlighting the underlying pharmacological effects (Table 1 and Table 2).

### 4.1. Preclinical Evidence of Kidney Protection by Black Cumin 

#### 4.1.1. Protection against Drug-Induced Kidney Injury 

Drug-induced nephrotoxicity is one of the most common causes of kidney injury. Methotrexate (MTX, a chemotherapeutic agent) increased MDA, and lessened GSH levels in kidney homogenate and *N. sativa* reversed its actions in nephrotoxic mice [14]. In parallel, it was shown that *N. sativa* in low concentrations could improve the efficacy and safety of MTX treatment in Wistar rats [58]. In a similar antioxidant mechanism, TQ ameliorated oxidative damage caused by another anticancer drug CP in rat kidneys [59]. In a subsequent study by this research group, administration of NSO (2 mL/kg bwt orally), before and after a single-dose CP treatment (6 mg/kg bwt. i.p), significantly attenuated the CP-induced increase in serum creatinine and blood urea nitrogen (BUN) and decreased in the activities of brush border membrane (BBM) enzymes in kidney cortical and medullary homogenates, as well as in isolated BBM vesicles (BBMV). These biochemical and histological data suggest a potential protective effect of NSO against CP-induced AKI [15]. In a similar study conducted by another team, *N. sativa* seed powder (NSP), extract (NSE), and NSO ameliorated the effects of CP-induced kidney toxicity in Sprague–Dawley rats by alleviating serum levels of urea, creatinine, and potassium, and a notable elevation of Na, Na/K, vitamin D, nutritional markers, and antioxidant enzymes [60]. Overall, these studies confirm that black cumin can be effective in minimizing toxic side effects, frequently encountered in cancer chemotherapy. These findings can be used to formulate a combined therapy that can effectively manage complications in cancer chemotherapy.

Penconazole is a widely used triazole fungicide in agriculture, human, and veterinary medicine. High doses of penconazole cause nephrotoxicity and kidney damage. The antioxidant properties of *N. sativa* could be attributed to ameliorate penconazole-induced nephrotoxicity in rats [21]. Differential treatments of black seed oil prevented and reversed haloperidol-induced nephrotoxicity by depleting K+, Na+, MDA contents, and aldose-reductase (AR) activity, and AMP hydrolysis with increased adenosine triphosphate (ATP) in rat kidneys [61]. The protective effects of *N. sativa* were evaluated on 4-Nonylphenol (4-NP)-induced nephrotoxicity in *Clarias gariepinus* fishes. The administration of *N. sativa* markedly minimized the nephrotoxic impact of 4-NP and maintained the normal kidney structure and function [62]. *N. sativa* has also been shown to be protective against nephrotoxicity caused by commonly used non-steroidal anti-inflammatory drugs such as acetylsalicylic acid [18], aspirin [17], and paracetamol [16] 

A study was designed to evaluate the kidney protective potential of NSO against thioacetamide (TAA)-induced nephrotoxicity in rats. The results implied that treatment of NSO significantly reversed TAA-elevated lipid profile, urea, creatinine, uric acid, sodium, and potassium levels in serum [63]. Accordingly, the combination of metformin and NSO showed ameliorative effects against TAA induced hepatorenal toxicity in rats [64]. 

#### 4.1.2. Protection against Heavy Metal-Induced Kidney Injury 

The kidney is the first target organ of heavy metal toxicity. Treatment of TQ and ebselen (Eb) inhibited arsenic-induced oxidative damage, apoptosis, and inflammation; and considerably attenuated arsenic accumulation in kidney tissues [19]. The suppressed immune responses in mice pretreated with the cadmium (Cd)-lead (Pb) mixture were reversed by *N. sativa* in the kidney of mice [65]. The nephroprotective potential of TQ in Cd toxicity might be due to its anti-oxidative and anti-apoptotic properties, which could be useful for achieving optimum effects [20]. These protective effects of black cumin against Pd-induced kidney injury were further supported by the research of Farrag and the team who reported that black seed treatment attenuated Pb-induced hepatorenal damage in male rats [66]. Similar antioxidant mechanisms, including induction of CAT, GPx, and glutathione reductase activities and increase in SOD and GSH levels, were involved in the kidney protective effect of TQ against Pb-induced kidney injury in rats [67].

#### 4.1.3. Protection against Insecticide-Induced Kidney Injury 

Diazinon is a commonly used pesticide to control pests. Diazinon-induced oxidative stress and kidney dysfunction in rats. Pretreatment of NSO markedly altered the diazinon-induced hepatotoxicity and nephrotoxicity [22]. Fipronil is a phenylpyrazole insecticide, widely used for agricultural and veterinary activities. TQ and diallyl sulfide protected against fipronil-induced oxidative kidney injury in rats [68].

#### 4.1.4. Protection against Chemical-Induced Kidney Injury 

Various chemicals can cause kidney injury. Oral administration of combined fish oil and NSO reduced carbon tetrachloride (CCl_4_)-induced liver and kidney injury in rats through exerting anti-inflammatory and antioxidant activity [69]. These findings were supported by a recent study which demonstrated that the administration of NSO exerted a protective effect on the brain, liver, and kidney during CCl_4_-induced oxidative stress [23].

NSO ameliorated sodium nitrite-induced nephrotoxicity through blocking oxidative stress, attenuating fibrosis and inflammation, restoring glycogen level, ameliorating cytochrome C oxidase, and inhibiting apoptosis [70]. Similarly, consumption of TQ (25 and 50 mg/kg, p.o., daily) showed protective effects against sodium nitrite-induced kidney toxicity in male rats through reducing oxidative stress, restoring the normal balance between pro- and anti-inflammatory cytokines, and protecting kidney tissue from extrinsic and intrinsic apoptosis [24], indicating that protective effects of NSO in the previous study were due to TQ-mediated antioxidant and anti-inflammatory effects. Besides, supplementation of NSO at 5 mL/kg body weight/dose/day for 28 days exerts a nephroprotective and diuretic activity by reducing considerably urinary and serum rates of calcium, phosphate and oxalate in Wistar rats [71], suggesting its protective effects against urolithiasis. 

#### 4.1.5. Protection against Renal Ischemia/Reperfusion Injury

Kidney ischemia-reperfusion injury (IRI) is a known model of acute kidney injury. Pretreatment with *N. sativa* has a protective effect against IRI-induced kidney damage by inhibiting apoptosis and cell proliferation [12]. This effect was further extended by TQ supplementation (10 mg/kg/day) which ameliorated the IRI effect on the hemodynamic and tubular kidney functional parameters as well as the expression of some kidney injury markers and pro-inflammatory and pro-fibrotic cytokines [13].

#### 4.1.6. Protection against Urolithiasis/Ureteral Obstruction

Unilateral ureteral obstruction (UUO) is a well-established experimental model of kidney fibrosis. UUO was related to a significant increase in oxidative stress, inflammation, and apoptosis [72]. *N. sativa* extract is a therapeutic agent to treat UUO-induced kidney damage comparable with captopril and losartan [72]. Similarly, TQ significantly improved oxidative damage, apoptosis, and TNF-α expression and markedly decreased the upregulation of angiotensin II and MCP-1 compared with the UUO rats [73]. 

#### 4.1.7. Protection against Other Stresses

Most chemotherapeutic drugs lead to nephrotoxicity. Experimental animal studies described the protective effect of TQ on chemotherapy-induced nephrotoxicity by decreasing lipid peroxidation and increasing the activity of antioxidant enzymes in the kidney tissue of chemotherapy-treated animals [74]. A preclinical in vitro study translated into better chemotherapeutics of TQ and its analogs to treat kidney cancer [75].

In acute kidney injury induced by sepsis in BALB/c mice, TQ administration through gavage reversed CLP-induced increase in serum levels of CRE and BUN and tissue expression of NLRP3, caspase-1, caspase-3, caspase-8, TNF-α, IL-1β, IL-6, and NF-κB, indicating that TQ may have a potential therapeutic benefit against sepsis-induced AKI [76]. LPS is also responsible for inducing sepsis-associated AKI [77,78]. TQ treatment reduced LPS-induced kidney fibrosis and permeability and improved oxidative stress status [79]. TQ exhibited protective effects on hyperuricemia-mediated kidney oxidative stress and mitochondrial abnormalities, which Nrf2/HO-1 could mediate, Akt signaling pathways [80]. Hypercholesterolemia is a well-established risk factor for kidney injury that can lead to CKD. NSO and TQ treatment reduced albuminuria in experimental rats of the streptozotocin (STZ)-induced diabetic nephropathy by preserving the podocyte function [81]. Another study suggests that TQ may be a potential therapeutic agent against kidney damage from hypercholesterolemia [82]. In addition, *N. sativa* ethanol extract treatment elevated nitric oxide (NO) levels and enlarged kidney arteriole diameter of a pre-eclampsia mouse model [83]. *N. sativa* and its components are also promising in preventing and curing nephrolithiasis and kidney damage [84].

### 4.2. Clinical Evidence of Kidney Protection by Black Cumin

Black cumin has shown improvement in disease outcomes in CKD patients as reported in several human studies. A recent systematic review and meta-analysis of randomized-controlled trials demonstrate that black cumin supplement in a long-term intervention and daily optimum dosage can significantly reduce parameters of kidney function, including BUN [85]. In a prospective, comparative, and open-labeled study on patients with CKD stages 3 and 4 at a tertiary care center in North India, treatment of NSO (2.5 mL, p.o., once daily for 12 weeks) significantly improved clinical features and biochemical parameters, including a reduction in blood urea, serum creatinine, and total urinary protein and an increase in total urine volume and glomerular filtration rate in 24 h [27]. Another similar study revealed the efficacy and safety of NSO administration in patients with CKD stages 3 and 4 due to diabetic nephropathy. There was a significant reduction in blood glucose, serum creatinine, blood urea, and 24 h total urinary protein levels and a significant increase in glomerular filtration rate, 24 h total urinary volume, and hemoglobin level in the group treated by black cumin oil [25]. In both studies, authors suggest that black cumin oil could be an add-on therapy that can boost the therapeutic advantage of conservative management in patients of diabetic nephropathy. Protection against nephrolithiasis as reproted in preclinical study [71] was further translated into a randomized, triple-blind, placebo-controlled, clinical trial in which two groups of patients (each with 30) with renal stones received either black seed capsules (500 mg) or placebo twice daily for 10 weeks. In the black seed group, 44.4% of patients excreted their stones completely, and the size of the stones remained unchanged and decreased in 3.7% and 51.8% of patients, respectively, while in the placebo group, 15.3% of the patients excreted their stones completely, 11.5% had a reduction in stone size, 15.3% had an increase in stone size, and 57.6% had no change in their stone size. There was a significant difference in the mean size of renal stones between the two groups. Compared to placebo, black seeds have strong positive effects on the disappearance or reducing the size of kidney stones [26]. 

**Table 1:** Summary on the protective effects of black cumin and TQ against various experimental kidney injury models.

Experimental Models	Treatment with Doses	Pathophysiological Alterations	Ref.
Acetylsalicylic acid-induced nephrotoxicity in rats	Ethanolic NSE (250 mg/kg)	Improved paired kidney weight, body weight, relative tissue body weight index, and normalized serum urea and creatinine	[18]
Aspirin-induced nephrotoxicity in rats	Ethanolic NSE (250 mg/kg)	Significant improvement in histological parameters, including disrupted brush border, epithelial necrosis, intraluminal protein casts, and basement membrane integrity	[17]
Calcium oxalate-induced urolithiasis in rats	NSO (5 mL/kg BW/dose/ day for 28 days)	↓Urinary and serum rates of calcium phosphate and oxalate; ↑volume of urine excreted	[71]
CCl_4_-induced kidney injury in rats	Combined fish oil/ NSO (300 mg oil emulsions /kg BW, for 20 days)	↑Unsaturated fatty acids; ↓oxidative stress and inflammation	[69]
CP-induced AKI in rats	NSO (2 mL/kg BW orally)	↓Serum creatinine, BUN and ↑BBM enzyme activities in kidney cortical and medullary homogenates and BBMV; carbohydrate metabolism enzyme activities, and in the enzymatic and non-enzymatic antioxidant parameters toward normalcy	[15]
CP-induced kidney toxicity in rats	NSP (3 g/kg/day), extract (0.5g/kg/ day) and NSO (2 g/kg/day) for 60 days	↓Serum levels of urea, creatinine, and K^+^; ↑Na^+^, Na^+^/K^+^ ratio, vitamin D, nutritional markers, and antioxidant enzymes	[60]
Diazinon-induced nephrotoxicity in rats	NSO (2 mg/kg/daily)	↓AST, ALT, ALP, BIL, creatinine and urea	[22]
Haloperidol (HAL)-induced nephrotoxicity in rats	NSO (Pre-, co- and post-treatment: 150 mg/kg BW for 7 days)	↓K^+^, Na^+^, MDA contents and aldose-reductase activity, and AMP hydrolysis; ↑ATP in the plasma cell membranes of rat kidney; ↓inner kidney cortex and outer medulla	[61]
IRI-induced kidney injury in rats	Single dose of NSP (400 mg/kg orally)	↓Stain-positive cells in kidney tissue; ↓tissue MDA levels; ↑GPx and CAT	[12]
Methotrexate-induced nephrotoxicity in mice	NSO (0.125 mL/daily)	↓MDA; ↑GSH levels in kidney homogenate	[14]
Paracetamol-induced nephrotoxicity in rats	Ethanolic NSE (250, 500 and 1000 mg/kg)	↓Serum urea and creatinine; ↑SOD and GSH; ↓MDA levels in the kidneys; reversed kidney pathological damage	[16]
Penconazole-induced nephrotoxicity in rats	NSO (orally 0.2ml black cumin oil /100 g BW three days/ week for four weeks)	↓Subcapsular space and hypercellularity of the glomerular cells; attachment of podocytes and their processes; ↑Bcl-2 immune marker; ↓intercalated cells of cortical; ↓α-SMA and collagen fibers; ↓MDA level; ↑SOD and CAT	[21]
Sodium nitrite-induced nephrotoxicity in rats	NSO (2.5, 5, and 10 mL/kg for 12 weeks)	↓Serum urea and creatinine; ↑normal appearance of kidney tissue; ↓glycogen levels; ↓fibrosis markers, partially; ↓caspase-3 and pJNK/JNK	[70]
Unilateral ureteral obstruction-induced kidney damage in rats	NSE (200 and 400 mg/kg, 2 doses for 18 days)	↓Kidney angiotensin II and monocyte chemoattractant protein-1 expression, MDA and TNF-α levels, and the number of apoptotic cells; ↑kidney total thiol content and the activity of antioxidant enzymes	[72]
Arsenic-induced kidney toxicity in female rats	TQ (10 mg/kg) and ebselen (5 mg/kg)	↓Oxidative stress, inflammation, apoptosis, As accumulation in the kidney tissue; ↓histological kidney damage	[19]
Cadmium-induced nephrotoxicity in rats	TQ (50 mg/kg BW)	↓Toxicity of Cd and preserved histological architecture of the kidney tissue; ↓Overexpression of NF-κB in kidney tissue; ↓apoptotic cells; subdued lipid peroxidation; ↓SOD, GPx, and CAT activities in kidney tissue	[20]
IRI-induced kidney injury in rats	TQ (10 mg/kg/day)	Reduction of IRI-related alteration in kidney functions: ↑left RBF and GFR; ↑left kidney FENa; ↓gene expressions of KIM-1, NGAL, TNF-α, TGF-β1 and PAI-1	[13]
Sodium nitrite-induced kidney toxicity in rats	TQ (25 and 50 mg/kg, p.o., daily)	↓Oxidative stress, restoration of pro- and anti-inflammatory cytokines and protection of kidney tissue from apoptosis	[24]
CP-induced nephrotoxicity in rats	NSO (2 mL/kg BW, orally) and TQ (1.5 mg/kg BW, orally)	Improve kidney function, restored serum creatinine and blood urea nitrogen levels; ↑BBM marker enzymes (ALP, GGTase and LAP) in BBMVs, homogenates of kidney cortex and medulla; ↓kidney metabolic and redox status	[59]

AKI, Acute kidney injury; ALP, Alkaline phosphatase; ALT, Alanine aminotransferase; AMP, Activated protein kinase; AST, Aspartate aminotransferase; ATP, Adenosine triphosphate, As, Arsenic; BBM, Brush border membrane; BBMV, Brush border membrane vesicle; BIL, Bilirubin; BUN, Blood urea nitrogen; Bcl-2, B-cell lymphoma 2; CAT, Catalase; CCl_4_, Carbon tetrachloride; CKD, Chronic kidney disease; CP, Cisplatin; Cd, Cadmium; FENa, Fractional excretion of sodium; GFR, Growth factor receptor; GGTase, Geranylgeranyltransferase; GPx, Glutathione peroxidase; GSH, Glutathione; IRI–Ischemia-reperfusion injury; JNK, c-Jun N-terminal kinases; KIM-1, Kidney injury molecule-1; LAP, latency-associated peptide; MDA, Malondialdehyde; NF-κB, Nuclear factor kappa B; NGAL, Neutrophil gelatinase-associated lipocalin; NSO, *N. sativa* oil; NSP, *N. sativa* seed powder; NSE, *N. sativa* seed extract; pJNK, Phosphorylated c-Jun N-terminal kinase; PAI-1, plasminogen activator inhibitor-1; RBF, Renal blood flow; SOD, Superoxide dismutase; TGF-β1, Transforming growth factor beta 1; TNF-α, Tumor necrosis factor alpha; TQ, Thymoquinone; α-SMA, Smooth muscle alpha-actin.

**Table 2:** Summary on the protective effects of black cumin against various kidney diseases in patients.

Types of Kidney Disease	Treatment with Doses	Pathophysiological Alterations	Ref.
Randomized, prospective, comparative, and open-labeled clinical trial with Stages 3 and 4 CKD patients	NSO (2.5 mL, p.o., once daily) along with alpha-keto analog of essential amino acids	↓Blood urea, serum creatinine, and 24-h total urine protein; ↑24-h total urine volume and glomerular filtration rate; delaying the progression of CKD at stages 3 and 4	[27]
Prospective, comparative, and open-label study with patients with CKD (Stage 3 and 4) due to diabetic nephropathy	NSO (2.5 mL, once daily and orally)	↓Blood glucose, serum creatinine, blood urea, 24 h total urinary protein levels; ↑glomerular filtration rate, 24 h total urinary volume, and hemoglobin level	[25]
Randomized, triple-blind, placebo-controlled, clinical trial in patients with kidney stones	Seed capsule (500 mg, twice for 10 weeks	Retreated or decreased the size of kidney stones	[26]

CKD, chronic kidney disease; NSO, *Nigella sativa* oil.

## 5. Safety Issues

Black cumin and its bioactive components are considered to be relatively well-tolerated [86,87]. However, TQ can, in some cases, generate oxidative stress, disrupt cellular macromolecules (DNA, lipids, and proteins) and signaling pathways, such as extracellular signal-regulated kinase (ERK), protein kinase C (PKC) and Ras, while other bioactive compounds from *N. sativa* can interfere with TQ-induced toxicity [88,89,90]. TQ toxicity is, however, context-dependent.

Administration of NSO up to 2.5 mL (orally, once daily) has been proven to be safe in terms of biochemical and clinical features of diabetic nephropathy patients, though several other molecular studies were required to confirm the statement [25]. It is also safe to take NSO (2.5 mL) three times a day as an add-on therapy to improve kidney function in CKD patients [27]. A randomized, double-blind, placebo-controlled clinical trial with *N. sativa* showed a significant decrease in kidney stone size, although one male out of 60 patients had hydronephrosis and raised blood pressure [26]. This study has limitations such as a shorter duration and the lack of specific tests (computed tomography) which were necessary to evaluate the size of kidney stones [26]. 

In one report, NSE (100 mg/kg) was administered two weeks after CP dosing, and no visible effects on kidney biochemical parameters were observed in rats [91]. Mice were given various doses of NSE (6, 9, 14, and 21 g/kg) and no mortality was reported [92]. In another experiment, NSO supplementation (0.2, 0.4, 0.4, 0.8, 1 mg/kg) to rats and mice resulted in zero mortality [93]. However, there were limitations in the characterization of NSO and no content other than the total flavonoid evaluation. A report on broiler chicks indicated that supplementation of 20 and 100 g/kg *N. sativa* seeds for 7 weeks had essentially no adverse effects on biochemical/hematological profile, pathological features, and growth [94]. Researchers proved that a higher dose (25 mL/kg) of NSO had toxic effects on the histological changes in the renal cortex and a lower dose (15 mL/kg) had a negative effect on the liver [95]. In addition, a previous study showed that TQ at higher than 10 mg/kg/day showed no protective effects against CP-induced nephrotoxicity [96]. Therefore, it suggests that appropriate doses of black cumin or its components are required to achieve the desired outcomes.

## 6. Concluding Remarks and Future Perspectives

**Figure 3:**
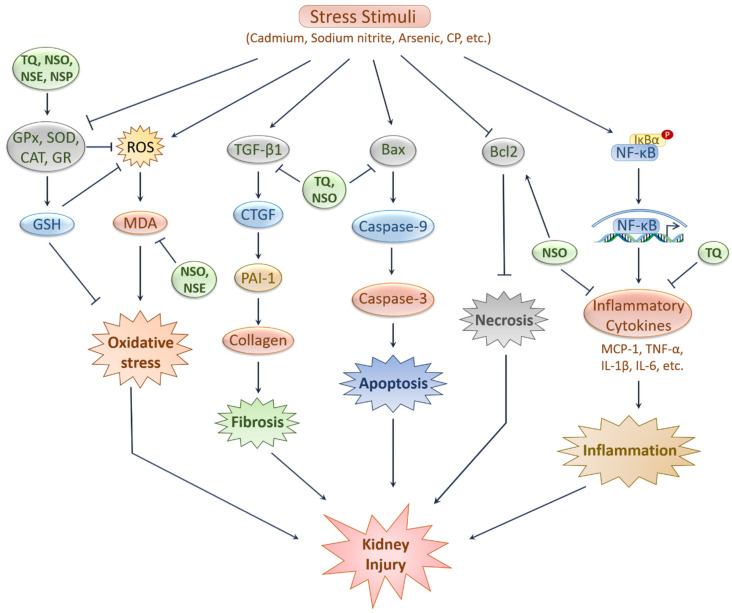
Prospective kidney-protective effects of N. sativa and its active constituent. Bioactive compounds of N. sativa prevents kidney injury by inhibiting several stress stimuli induced apoptosis, oxidative stress, inflammation and fibrosis. Stress stimuli such as cadmium, CP, sodium nitrite, and so forth causes oxidative stress by lessening the antioxidant enzymes and elevating the level of ROS and MDA. TQ, NSO, NSE and NSP elevate the antioxidant enzymes leading to increased GSH and reduced ROS level. NSO and NSE also reduce MDA level to prevent oxidative stress. TQ and NSO prevent stress stimuli induced fibrosis by downregulating fibrosis markers, such as TGF-β1, PAI-1, and collagen. They also prevent apoptosis by reducing apoptosis-related markers, such as Bax and caspase-3. NSO attenuates necrosis through upregulating Bcl-2. TQ and NSO ameliorate inflammation by lessening inflammatory cytokines, such as TNF-α, IL-1β and IL-6. Bax, Bcl-2-associated X protein; Bcl2, B-cell lymphoma 2; CAT, Catalase; CP, Cisplatin; GPx, Glutathione peroxidase; GR, Glutathione reductase; GSH, Glutathione; IL-1β, Interleukin-1 beta; IL-6, Interleukin-6; MCP-1, Monocyte Chemoattractant Protein-1; MDA, Malondialdehyde; NF-κB, Nuclear factor kappa B; NSE, N. sativa extract; NSO, N. sativa oil; NSP, N. sativa powder; PAI-1, Plasminogen activator inhibitor-1; SOD, Superoxide dismutase; TGF-β1, Transforming growth factor beta 1; TNF-α, Tumor necrosis factor alpha; TQ, Thymoquinone.

Kidneys are constantly exposed to various xenobiotics, namely drugs, food additives, poisons, and environmental chemicals. The damaging effects of these xenobiotics severely restrict kidney function and lead to the development of acute as well as chronic kidney diseases. There are some other predisposing factors, such as diabetes, hypertension, dyslipidemia, and ischemia that also increase the risk of kidney disease. The multiple side effects posed by the existing therapeutic agents motivate scientists to explore safer alternatives. Evidence from the existing literature suggests that exposure to xenobiotics, namely chemotherapeutics, heavy metals, pesticides, and other environmental chemicals causes kidney injury in experimental animals, which was improved by the administration of black cumin and TQ (Figure 3). Ischemia/reperfusion can also damage kidneys, which can be treated with black cumin and TQ. Moreover, there is ample evidence that black cumin and TQ can also improve kidney conditions in experimental diabetes and other complications [97]. The plausible mechanisms underlying the protective effects of black cumin and TQ against various kidney complications primarily involve antioxidation, anti-inflammation, anti-apoptosis, and antifibrosis. While existing evidence suggests that NF-κB, Caspase, and TGF-β signaling pathways are involved as the underlying molecular mechanisms of black cumin/TQ-mediated kidney protective effects, it is necessary to investigate whether some other important pathways such as Nrf2/HO-1, mTOR, MAPK are implicated. 

The preclinical outcomes have also been translated into clinical subjects as there is evidence that black cumin oil given to advanced CKD patients normalizes hematological and urinary parameters and improves disease outcomes. However, in order to recommend this natural remedy in patients with kidney complications, further clinical studies with appropriate human subjects, and longer duration are warranted. Information on nanoparticle-guided targeted delivery in the kidney is also lacking. However, this review provides some valuable information that scientists can use to advance future research into black cumin/TQ-based therapies against kidney disease.

## Data Availability

Not applicable.

## References

[1] Uchino S., Kellum J.A., Bellomo R., Doig G.S., Morimatsu H., Morgera S., Schetz M., Tan I., Bouman C., Macedo E. (2005). Acute renal failure in critically ill patients: A multinational, multicenter study. JAMA.

[2] Coca S.G., Singanamala S., Parikh C.R. (2012). Chronic kidney disease after acute kidney injury: A systematic review and meta-analysis. Kidney Int..

[3] Lewington A.J., Cerda J., Mehta R.L. (2013). Raising awareness of acute kidney injury: A global perspective of a silent killer. Kidney Int..

[4] Doi K. (2016). Role of kidney injury in sepsis. J. Intensive Care.

[5] Togel F., Westenfelder C. (2014). Recent advances in the understanding of acute kidney injury. F1000Prime Rep..

[6] Radi Z.A. (2019). Kidney pathophysiology, toxicology, and drug-induced injury in drug development. Int. J. Toxicol..

[7] Dixon J., Lane K., Macphee I., Philips B. (2014). Xenobiotic metabolism: The effect of acute kidney injury on non-renal drug clearance and hepatic drug metabolism. Int. J. Mol. Sci..

[8] Kinsey G.R., Okusa M.D. (2011). Pathogenesis of acute kidney injury: Foundation for clinical practice. Am. J. Kidney Dis..

[9] Kooti W., Hasanzadeh-Noohi Z., Sharafi-Ahvazi N., Asadi-Samani M., Ashtary-Larky D. (2016). Phytochemistry, pharmacology, and therapeutic uses of black seed (*Nigella sativa*). Chin. J. Nat. Med..

[10] Kanter M., Coskun O., Uysal H. (2006). The antioxidative and antihistaminic effect of *Nigella sativa* and its major constituent, thymoquinone on ethanol-induced gastric mucosal damage. Arch. Toxicol..

[11] Hannan M.A., Rahman M.A., Sohag A.A.M., Uddin M.J., Dash R., Sikder M.H., Rahman M.S., Timalsina B., Munni Y.A., Sarker P.P. (2021). Black Cumin (*Nigella sativa* L.): A Comprehensive Review on Phytochemistry, Health Benefits, Molecular Pharmacology, and Safety. Nutrients.

[12] Caskurlu T., Kanter M., Erboga M., Erboga Z.F., Ozgul M., Atis G. (2016). Protective effect of Nigella Sativa on renal reperfusion injury in rat. Iran. J. Kidney Dis..

[13] Hammad F.T., Lubbad L. (2016). The effect of thymoquinone on the renal functions following ischemia-reperfusion injury in the rat. Int. J. Physiol. Pathophysiol. Pharm..

[14] Ahmed J.H., Abdulmajeed I.M. (2017). Effect of *Nigella sativa* (black seeds) against methotrexate-induced nephrotoxicity in mice. J. Intercult. Ethnopharmcol..

[15] Farooqui Z., Ahmed F., Rizwan S., Shahid F., Khan A.A., Khan F. (2017). Protective effect of *Nigella sativa* oil on cisplatin induced nephrotoxicity and oxidative damage in rat kidney. Biomed. Pharmacother..

[16] Canayakin D., Bayir Y., Kilic Baygutalp N., Sezen Karaoglan E., Atmaca H.T., Kocak Ozgeris F.B., Keles M.S., Halici Z. (2016). Paracetamol-induced nephrotoxicity and oxidative stress in rats: The protective role of *Nigella sativa*. Pharm. Biol..

[17] Asif S., Mudassir S., Toor R.S. (2018). Histological Effects of *Nigella sativa* on Aspirin-Induced Nephrotoxicity in Albino Rats. J. Coll. Physicians Surg. Pak..

[18] Asif S., Malik L. (2017). Protective effects of *Nigella sativa* on acetylsalicylic acid-induced nephrotoxicity in albino rats. J. Coll. Physicians Surg. Pak..

[19] Al-Brakati A.Y., Kassab R.B., Lokman M.S., Elmahallawy E.K., Amin H.K., Abdel Moneim A.E. (2019). Role of thymoquinone and ebselen in the prevention of sodium arsenite–induced nephrotoxicity in female rats. Hum. Exp. Toxicol..

[20] Erboga M., Kanter M., Aktas C., Sener U., Fidanol Erboga Z., Bozdemir Donmez Y., Gurel A. (2016). Thymoquinone Ameliorates Cadmium-Induced Nephrotoxicity, Apoptosis, and Oxidative Stress in Rats is Based on its Anti-Apoptotic and Anti-Oxidant Properties. Biol. Trace Elem. Res..

[21] Khair N.S., Nooreldien N.M. (2019). The protective effect of *Nigella sativa* oil on Penconazole induced -renal toxicity in adult albino rats: Histological, Immunohistochemical and Biochemical study. Egypt. J. Histol..

[22] Alhilo R.M., Kadhim H.J., Abbas M.T. (2019). Effects of nigella sativa oil on biochemical parameters of white male rats exposed to diazinon. Indian J. Public Health Res. Dev..

[23] Ebuehi O.A.T., Olowojaiye A.A., Erukainure O.L., Ajagun-Ogunleye O.M. (2020). *Nigella sativa* (black seed) oil ameliorates CCl4-induced hepatotoxicity and mediates neurotransmitter levels in male Sprague Dawley albino rats. J. Food Biochem..

[24] Elsherbiny N.M., Maysarah N.M., El-Sherbiny M., Al-Gayyar M.M. (2017). Renal protective effects of thymoquinone against sodium nitrite-induced chronic toxicity in rats: Impact on inflammation and apoptosis. Life Sci..

[25] Ansari Z.M., Nasiruddin M., Khan R.A., Haque S.F. (2017). Protective role of *Nigella sativa* in diabetic nephropathy: A randomized clinical trial. Saudi J. Kidney Dis. Transpl..

[26] Ardakani Movaghati M.R., Yousefi M., Saghebi S.A., Sadeghi Vazin M., Iraji A., Mosavat S.H. (2019). Efficacy of black seed (*Nigella sativa* L.) on kidney stone dissolution: A randomized, double-blind, placebo-controlled, clinical trial. Phytother. Res..

[27] Alam M.A., Nasiruddin M., Haque S.F., Khan R.A. (2020). Evaluation of safety and efficacy profile of *Nigella sativa* oil as an add-on therapy, in addition to alpha-keto analogue of essential amino acids in patients with chronic kidney disease. Saudi J. Kidney Dis. Transpl..

[28] Ahmad A., Husain A., Mujeeb M., Khan S.A., Najmi A.K., Siddique N.A., Damanhouri Z.A., Anwar F. (2013). A review on therapeutic potential of *Nigella sativa*: A miracle herb. Asian Pac. J. Trop. Biomed..

[29] Islam M.N., Hossain K.S., Sarker P.P., Ferdous J., Hannan M.A., Rahman M.M., Chu D.T., Uddin M.J. (2021). Revisiting pharmacological potentials of *Nigella sativa* seed: A promising option for COVID-19 prevention and cure. Phytother. Res..

[30] Uddin M.J., Kim E.H., Hannan M.A., Ha H. (2021). Pharmacotherapy against oxidative stress in chronic kidney disease: Promising small molecule natural products targeting nrf2-ho-1 signaling. Antioxidants.

[31] Pandiri I., Moni A. (2018). Ocimum herb species: A potential treatment strategy for diabetic kidney disease. J. Adv. Biotechnol. Exp. Ther..

[32] Farjana M., Moni A., Sohag A.A.M., Hasan A., Hannan M.A., Hossain M.G., Uddin M.J. (2020). Repositioning vitamin C as a promising option to alleviate complications associated with COVID-19. Infect. Chemother..

[33] Moni A., Iqbal A., Uddin M. (2018). Resveratrol attenuates inflammation through tristetraprolin expression in human hepatocytes. J. Adv. Biotechnol. Exp. Ther..

[34] Hassanien M.F., Assiri A.M., Alzohairy A.M., Oraby H.F. (2015). Health-promoting value and food applications of black cumin essential oil: An overview. J. Food Sci. Technol..

[35] Yimer E.M., Tuem K.B., Karim A., Ur-Rehman N., Anwar F. (2019). *Nigella sativa* L. (black cumin): A promising natural remedy for wide range of illnesses. Evid.-Based Complement. Altern. Med..

[36] Omidi H., Khorram S., Mesgari M., Asghari-Jafarabadi M., Tarighat-Esfanjani A. (2017). Effects of separate and concurrent supplementation of Nano-sized clinoptilolite and *Nigella sativa* on oxidative stress, anti-oxidative parameters and body weight in rats with type 2 diabetes. Biomed. Pharm..

[37] Ozdemir N., Kantekin-Erdogan M.N., Tat T., Tekin A. (2018). Effect of black cumin oil on the oxidative stability and sensory characteristics of mayonnaise. J. Food Sci. Technol..

[38] Sultan M.T., Butt M.S., Karim R., Ahmed W., Kaka U., Ahmad S., Dewanjee S., Jaafar H.Z., Zia-Ul-Haq M. (2015). Nigella sativa fixed and essential oil modulates glutathione redox enzymes in potassium bromate induced oxidative stress. BMC Complement. Altern. Med..

[39] Mostafa R.M., Moustafa Y.M., Mirghani Z., AlKusayer G.M., Moustafa K.M. (2013). Antioxidant effect of garlic (*Allium sativum*) and black seeds (*Nigella sativa*) in healthy postmenopausal women. SAGE Open Med..

[40] Kazemi M. (2014). Phytochemical composition, antioxidant, anti-inflammatory and antimicrobial activity of *Nigella sativa* L. essential oil. J. Essent. Oil Bear. Plants.

[41] Singh S., Das S.S., Singh G., Schuff C., de Lampasona M.P., Catalán C.A.N. (2014). Composition, in vitro antioxidant and antimicrobial activities of essential oil and oleoresins obtained from black cumin seeds (*Nigella sativa* L.). BioMed. Res. Int..

[42] Imam A., Sulaiman N., Oyewole A., Amin A., Shittu S., Ajao M. (2018). Pro-neurogenic and antioxidant efficacy of *Nigella sativa* oil reduced vulnerability to cholinesterase dysfunction and disruption in amygdala-dependent behaviours in chlorpyrifos exposure. J. Krishna Inst. Med. Sci. Univ..

[43] Mabrouk A. (2017). Protective effect of thymoquinone against lead-induced antioxidant defense system alteration in rat liver. Acta Biol. Hung..

[44] Cobourne-Duval M.K., Taka E., Mendonca P., Bauer D., Soliman K.F. (2016). The Antioxidant Effects of Thymoquinone in Activated BV-2 Murine Microglial Cells. Neurochem. Res..

[45] El-Gindy Y., Zeweil H., Zahran S., El-Rahman M.A., Eisa F. (2020). Hematologic, lipid profile, immunity, and antioxidant status of growing rabbits fed black seed as natural antioxidants. Trop. Anim. Health Prod..

[46] Shahid F., Farooqui Z., Khan A.A., Khan F. (2018). Oral *Nigella sativa* oil and thymoquinone administration ameliorates the effect of long-term cisplatin treatment on the enzymes of carbohydrate metabolism, brush border membrane, and antioxidant defense in rat intestine. Naunyn-Schmiedebergs Arch. Pharm..

[47] Ardiana M., Pikir B.S., Santoso A., Hermawan H.O., Al-Farabi M.J. (2020). Effect of *Nigella sativa* supplementation on oxidative stress and antioxidant parameters: A meta-analysis of randomized controlled trials. Sci. World J..

[48] Namazi N., Mahdavi R., Alizadeh M., Farajnia S. (2015). Oxidative Stress Responses to *Nigella sativa* Oil Concurrent with a Low-Calorie Diet in Obese Women: A Randomized, Double-Blind Controlled Clinical Trial. Phytother. Res..

[49] Dwita L.P., Yati K., Gantini S.N. (2019). The anti-inflammatory activity of *Nigella sativa* balm sticks. Sci. Pharm..

[50] Koshak A., Koshak E., Heinrich M. (2017). Medicinal benefits of *Nigella sativa* in bronchial asthma: A literature review. Saudi Pharm. J..

[51] Hossen M.J., Yang W.S., Kim D., Aravinthan A., Kim J.-H., Cho J.Y. (2017). Thymoquinone: An IRAK1 inhibitor with in vivo and in vitro anti-inflammatory activities. Sci. Rep..

[52] Houghton P.J., Zarka R., de las Heras B., Hoult J.R. (1995). Fixed oil of *Nigella sativa* and derived thymoquinone inhibit eicosanoid generation in leukocytes and membrane lipid peroxidation. Planta Med..

[53] Mansour M., Tornhamre S. (2004). Inhibition of 5-lipoxygenase and leukotriene C4 synthase in human blood cells by thymoquinone. J. Enzym. Inhib. Med. Chem..

[54] Aziz N., Son Y.J., Cho J.Y. (2018). Thymoquinone suppresses IRF-3-mediated expression of type I interferons via suppression of TBK1. Int. J. Mol. Sci..

[55] Abbas A.T., Abdel-Aziz M.M., Zalata K.R., Abd Al-Galel Tel D. (2005). Effect of dexamethasone and *Nigella sativa* on peripheral blood eosinophil count, IgG1 and IgG2a, cytokine profiles and lung inflammation in murine model of allergic asthma. Egypt J. Immunol..

[56] Attia H.N., Ibrahim F.M., Maklad Y.A., Ahmed K.A., Ramadan M.F. (2016). Characterization of antiradical and anti-inflammatory activities of some cold pressed oils in carrageenan-induced rat model of acute inflammation. Der. Pharma Chem..

[57] Bordoni L., Fedeli D., Nasuti C., Maggi F., Papa F., Wabitsch M., De Caterina R., Gabbianelli R. (2019). Antioxidant and anti-inflammatory properties of *Nigella sativa* oil in human pre-adipocytes. Antioxidants.

[58] Ahmed E., Abd-ellatief R., Ali M., Saleh T., Ahmed E. (2020). Optimization of the effectiveness and cytocompatibility of *Nigella sativa* as a co-treatment for reducing methotrexate-related adverse effects. Comp. Clin. Pathol..

[59] Farooqui Z., Shahid F., Abidi S., Parwez I., Khan F. (2017). Oral thymoquinone administration ameliorates: The effect of cisplatin on brush border membrane enzymes, energy metabolism, and redox status in rat kidney. Naunyn-Schmiedebergs Arch. Pharmacol..

[60] Alsuhaibani A.M.A. (2018). Effect of *Nigella sativa* against cisplatin induced nephrotoxicity in rats. Ital. J. Food Saf..

[61] Akintunde J.K., Abubakar O.K. (2017). Novel therapeutic approaches of natural oil from black seeds and its underlying mechanisms against kidney dysfunctions in haloperidol-induced male rats. Drug Metab. Pers. Ther..

[62] Kotb A.M., Abd-Elkareem M., Abou Khalil N.S., Sayed A.E.D.H. (2018). Protective effect of *Nigella sativa* on 4-nonylphenol-induced nephrotoxicity in Clarias gariepinus (Burchell, 1822). Sci. Total Environ..

[63] Ahmad A., Al-Abbasi F., Sadath S., Ali S.S., Abuzinadah M.F., Alhadrami H.A., Alghamdi A.A.M., Aseeri A.H., Khan S., Husain A.A. (2018). Ameliorative effect of camel’s milk and *Nigella sativa* Oil against thioacetamide-induced hepatorenal damage in rats. Pharmacogn. Mag..

[64] Ahmad A., Alkreathy H.M. (2018). Comparative biochemical and histopathological studies on the efficacy of metformin and *Nigella sativa* oil against thioacetamide-induced acute hepatorenal damage in rats. Biomed. Res..

[65] Massadeh A.M., Al-Safi S.A., Momani I.F., Al-Mahmoud M., Alkofahi A.S. (2007). Analysis of cadmium and lead in mice organs: Effect of *Nigella sativa* L. (Black Cumin) on the distribution and immunosuppressive effect of cadmium-lead mixture in mice. Biol. Trace Elem. Res..

[66] Farrag A.R., Mahdy K.A., Abdel Rahman G.H., Osfor M.M. (2007). Protective effect of *Nigella sativa* seeds against lead-induced hepatorenal damage in male rats. Pak. J. Biol. Sci..

[67] Mabrouk A., Cheikh H.B. (2016). Thymoquinone ameliorates lead-induced suppression of the antioxidant system in rat kidneys. Libyan J. Med..

[68] Abdel-Daim M.M., Shaheen H.M., Abushouk A.I., Toraih E.A., Fawzy M.S., Alansari W.S., Aleya L., Bungau S. (2018). Thymoquinone and diallyl sulfide protect against fipronil-induced oxidative injury in rats. Environ. Sci. Pollut. Res. Int..

[69] Al-Okbi S.Y., Mohamed D.A., Hamed T.E., Edris A.E., Fouda K. (2018). Hepatic regeneration and reno-protection by fish oil, *Nigella sativa* oil and combined fish oil/Nigella sativa volatiles in CCL4 treated rats. J. Oleo Sci..

[70] Al-Gayyar M.M.H., Hassan H.M., Alyoussef A., Abbas A., Darweish M.M., El-Hawwary A.A. (2016). *Nigella sativa* oil attenuates chronic nephrotoxicity induced by oral sodium nitrite: Effects on tissue fibrosis and apoptosis. Redox Rep..

[71] Benhelima A., Kaid-Omar Z., Hemida H., Benmahdi T., Addou A. (2016). Nephroprotective and diuretic effect of *Nigella sativa* L seeds oil on lithiasic wistar rats. Afr. J. Trad. Complement. Altern. Med..

[72] Hosseinian S., Ebrahimzadeh Bideskan A., Shafei M.N., Sadeghnia H.R., Soukhtanloo M., Shahraki S., Samadi Noshahr Z., Khajavi Rad A. (2018). *Nigella sativa* extract is a potent therapeutic agent for renal inflammation, apoptosis, and oxidative stress in a rat model of unilateral ureteral obstruction. Phytother. Res..

[73] Hosseinian S., Rad A.K., Bideskan A.E., Soukhtanloo M., Sadeghnia H., Shafei M.N., Motejadded F., Mohebbati R., Shahraki S., Beheshti F. (2017). Thymoquinone ameliorates renal damage in unilateral ureteral obstruction in rats. Pharm. Rep..

[74] Cascella M., Palma G., Barbieri A., Bimonte S., Amruthraj N.J., Muzio M.R., del Vecchio V., Rea D., Falco M., Luciano A. (2017). Role of *Nigella sativa* and its constituent thymoquinone on chemotherapy-induced nephrotoxicity: Evidences from experimental animal studies. Nutrients.

[75] Dera A., Rajagopalan P. (2019). Thymoquinone attenuates phosphorylation of AKT to inhibit kidney cancer cell proliferation. J. Food Biochem..

[76] Guo L.-P., Liu S.-X., Yang Q., Liu H.-Y., Xu L.-L., Hao Y.-H., Zhang X.-Q. (2020). Effect of Thymoquinone on Acute Kidney Injury Induced by Sepsis in BALB/c Mice. BioMed Res. Int..

[77] Uddin M.J., Dorotea D., Pak E.S., Ha H. (2020). Fyn kinase: A potential therapeutic target in acute kidney injury. Biomol. Ther..

[78] Uddin M.J., Pak E.S., Ha H. (2018). Carbon monoxide releasing molecule-2 protects mice against acute kidney injury through inhibition of ER stress. Korean J. Physiol. Pharm..

[79] Bargi R., Asgharzadeh F., Beheshti F., Hosseini M., Farzadnia M., Khazaei M. (2017). Thymoquinone protects the rat kidneys against renal fibrosis. Res. Pharm. Sci..

[80] Dera A.A., Rajagopalan P., Alfhili M.A., Ahmed I., Chandramoorthy H.C. (2020). Thymoquinone attenuates oxidative stress of kidney mitochondria and exerts nephroprotective effects in oxonic acid-induced hyperuricemia rats. BioFactors.

[81] Al-Trad B., Al-Batayneh K., El-Metwally S., Alhazimi A., Ginawi I., Alaraj M., Alkofahi E., Aljumaili O., Kosba A. (2016). *Nigella sativa* oil and thymoquinone ameliorate albuminuria and renal extracellular matrix accumulation in the experimental diabetic rats. Eur. Rev. Med. Pharm. Sci..

[82] Pei Z., Zhu L., Liu Y., Li N., Yang G., Liu H. (2017). Thymoquinone reduces kidney damage in apolipoprotein E-deficient mice fed a high-cholesterol diet. RSC Adv..

[83] Purnamayanti N.M.D., Windu S.C., Poeranto S. (2018). Effect of *Nigella sativa* ethanol extract on the nitric oxide content and renal arteriole diameter of a pre-eclampsia mouse model. Eurasian J. Med..

[84] Hayatdavoudi P., Khajavi Rad A., Rajaei Z., Hadjzadeh M.A. (2016). Renal injury, nephrolithiasis and *Nigella sativa*: A mini review. Avicenna J. Phytomed..

[85] Razmpoosh E., Safi S., Abdollahi N., Nadjarzadeh A., Nazari M., Fallahzadeh H., Mazaheri M., Salehi-Abargouei A. (2020). The effect of *Nigella sativa* on the measures of liver and kidney parameters: A systematic review and meta-analysis of randomized-controlled trials. Pharmacol. Res..

[86] Ali B.H., Blunden G. (2003). Pharmacological and toxicological properties of *Nigella sativa*. Phytother. Res..

[87] Mashayekhi-Sardoo H., Rezaee R., Karimi G. (2020). An overview of in vivo toxicological profile of thymoquinone. Toxin Rev..

[88] Bolton J.L., Trush M.A., Penning T.M., Dryhurst G., Monks T.J. (2000). Role of quinones in toxicology. Chem. Res. Toxicol..

[89] El-Hadiyah T., Raza M., Mohammed O.Y., Abdallah A.A. (2003). Evaluation of *Nigella sativa* seed constituents for their in vivo toxicity in mice. Nat. Prod. Sci..

[90] Yu S.M., Kim S.J. (2015). The thymoquinone-induced production of reactive oxygen species promotes dedifferentiation through the ERK pathway and inflammation through the p38 and PI3K pathways in rabbit articular chondrocytes. Int. J. Mol. Med..

[91] El Daly E.S. (1998). Protective effect of cysteine and vitamin E, Crocus sativus and *Nigella sativa* extracts on cisplatin-induced toxicity in rats. J. Pharm. Belg..

[92] Vahdati-Mashhadian N., Rakhshandeh H., Omidi A. (2005). An investigation on LD50 and subacute hepatic toxicity of Nigella sativa seed extracts in mice. Die Pharm..

[93] Amina B. (2016). Toxicity and anti-oxidant activity of the essential oil of *Nigella sativa*. Der Pharm. Lett..

[94] Al-Homidan A., Al-Qarawi A., Al-Waily S., Adam S. (2002). Response of broiler chicks to dietary Rhazya stricta and Nigella sativa. Br. Poult. Sci..

[95] Zaghlol D., Kamel E., Mohammed D., Abbas N. (2012). The possible toxic effect of different doses of *Nigella sativa* oil on the histological structure of the liver and renal cortex of adult male albino rats. Egypt. J. Histol..

[96] Badary O.A., Nagi M.N., al-Shabanah O.A., al-Sawaf H.A., al-Sohaibani M.O., al-Bekairi A.M. (1997). Thymoquinone ameliorates the nephrotoxicity induced by cisplatin in rodents and potentiates its antitumor activity. Can. J. Physiol. Pharmacol..

[97] Shaterzadeh-Yazdi H., Noorbakhsh M.F., Samarghandian S., Farkhondeh T. (2018). An Overview on Renoprotective Effects of Thymoquinone. Kidney Dis..

